# Validating the Cattell-Braasch maneuver with a case of inherent atypical malrotation of the bowel: a case report

**DOI:** 10.1186/s13256-024-04869-6

**Published:** 2024-11-12

**Authors:** Athanasios Alvanos, David Junk, Ingo Bechmann, Hanno Steinke

**Affiliations:** Faculty of Medicine, Institute of Anatomy, University of Leipzig, Liebigstraße 13, 04103 Leipzig, Germany

**Keywords:** Atypical malrotation, Embryology, Cattell-Braasch maneuver, Surgical oncology, Case report

## Abstract

**Background:**

Malrotation of the bowel due to imperfect embryologic development is a rare condition with a wide spectrum of resulting anatomical variations. Similar conditions are achieved in the adult by derotating the bowel via the Cattell-Braasch maneuver. However, possible preparational bias might compromise the resulting topography.

**Case presentation:**

We present a case of atypical malrotation of the bowel in a cadaver study using a 96-year-old Caucasian male specimen with incidental finding of the pathology post mortem with no known surgical intervention in the abdomen during his lifetime. We compare the topography and abdominal layers with the anatomy of a 98-year-old Caucasian female specimen where the Cattell-Braasch maneuver was used to revert the embryologic development.

**Conclusions:**

Reverting the embryologic development in the adult via Cattell-Braasch maneuver enables to mirror inherent malrotation and reestablishes the position of the bowel prior to its rotation. The Cattell-Braasch maneuver is further validated in this study by showing that it is able to demonstrate essential layers for surgical interventions without damaging their integrity. Atypical malrotation unmasks those fascial border-like layers, which are often hidden due to adhesions and fusing of tissue during the usual embryologic development. Developmental defects present a chance to explore essential surgical layers that are otherwise masked by artifacts due to fusion of layers of connective tissue.

## Background

The embryologic development of the gut unfolds in several stages, beginning with a differentiation into foregut, midgut, and hindgut in the fourth week post fertilization [1, 2]. The following proliferational and rotational steps extend from about 30 days post fertilization until the end of the embryonic phase [1–4]. In a first step, we encounter a physiological herniation of the primitive gut into the extraembryonic coelom. The first stage includes a counterclockwise rotation of the herniated gut by 90°, resulting in a now horizontal orientation [1, 2, 4]. A further rotation by 180° occurs in a second step, which results in the large intestine forming the frame around the small intestine after reposition in the abdominal cavity. In the final steps, proliferation of the left-sided cecum places it in the lower-right abdominal quadrant [1, 4]. In their final position, the ascending and descending colon “fuse” with the back wall of the abdominal cavity [4].

According to Adams et al., malrotation of the bowel appears in 1 out of 500 people [5]. A wide spectrum of malrotation is distinguished, depending on the extent of the insufficiency of the described steps while specifically taking the resulting position of the cecum and duodenum into account [4, 6–9]. An essential differentiation appears to concern the position of the ligament of Treitz in relation to the midline and the level of the pylorus. While in “typical” cases of malrotation the ligament of Treitz is either absent or located on the right side of the midline, in atypical malrotation the ligament of Treitz is located on the left side of the midline and below the level of the pyloric outlet compared with the normal anatomy [7, 8].

Atypical malrotation has a significantly lower risk for the development of a volvulus, which rationalizes not rushing to surgical correction but favoring a conservative approach in asymptomatic patients [7, 8]. This, however, is a simplification of a very complex decision-making process due to the plethora of anatomical variations and clinical considerations.

In this case report, we present the anatomy in a human specimen in which an atypical malrotation was encountered in our dissection course and compare it with the surgical Cattell-Braasch maneuver, which reverts the embryologic development by surgical derotation of the bowel [10, 11].

The method is susceptible to preparational bias, which leaves unanswered the question of whether the adhesions that are released and the layers that are thus presented are valid and anatomically sound. We use this rare case of atypical malrotation to evaluate the Cattell-Braasch maneuver and the resulting anatomy.

## Case presentation

We present the anatomy of a 96-year-old Caucasian male human specimen. For demonstrational purposes, we have added images of a 98-year-old Caucasian female specimen to present the Cattell-Braasch maneuver in direct comparison.

Permission for educational and scientific use at the Institute of Anatomy at the University of Leipzig was granted by the body donors before death in accordance with the Saxonian Death and Funeral Act of 1994. The study was conducted in accordance with the Declaration of Helsinki, and approval by the institutional review board of the University of Leipzig was granted for anatomical studies on body donations after informed consent (project identification code 129/21-ek, date of approval 1/3/2022). The signed consent documents can be provided by the corresponding author on reasonable request. The body of the male donor was prepared for our dissection course using ethanol–glycerin fixation with thymol conservation [12]. The female body donor was used without fixation shortly after death.

In our presented case, a detailed patient history is lacking owing to the incidental finding post mortem, especially regarding recurrent abdominal symptoms. However, we did not observe any scars and encountered no peritoneal adhesions that might have been caused by surgical intervention. The cause of death was a stroke, according to the death certificate.

**Fig. 1:**
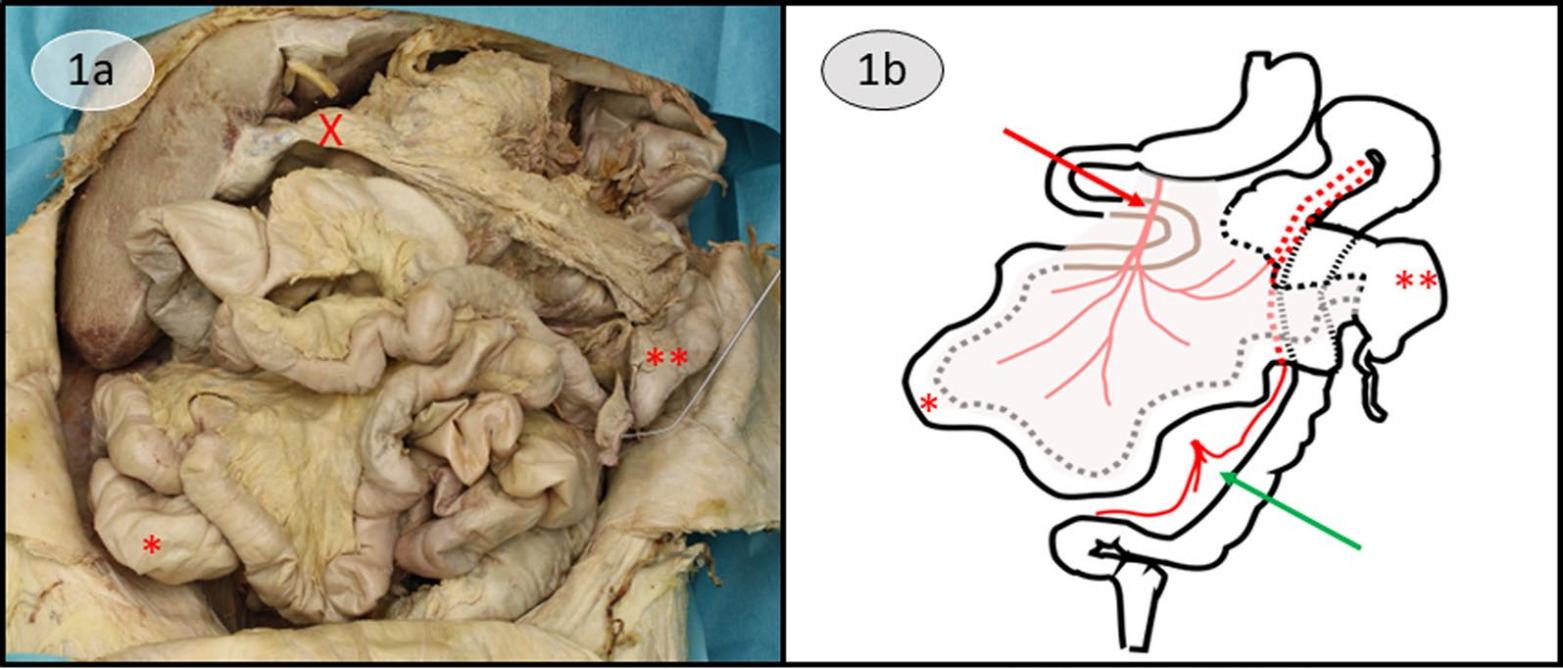
View onto the abdominal cavity after inverted Y-incision. **a** Intraperitoneal anatomy after inverted Y-incision shows no pathology besides diverticulosis of the sigmoid colon (not shown here) and inflammatory adhesion of the greater omentum on the gallbladder (X). The small intestine (*) is located in the right hemiabdomen, the large intestine with the cecum (**) in the left hemiabdomen. The instrument aims at the appendix. **b** Schematic depiction of the atypical malrotation at hand. The course of the duodenum and the duodenojejunal loop (brown) behind the mesenteric root with the superior mesenteric artery (red arrow) is outlined in the schematic draft. The dotted gray line shows where the meso (pale overlay) is attached to the small intestine, the dotted black line shows the attachment to the cecum (**) and following part of the large intestine. The dotted red line shows the anastomosis of Riolan connecting the superior mesenteric artery with the inferior mesenteric artery (green arrow). Position of the bowel mirrors the position in Fig. 2a

After opening the abdomen with an inverted Y-incision, we encountered the anatomy as depicted in Fig. 1. The abdominal organs showed no visible sign of tumorous growth. The only intraabdominal pathologies that were observed included diverticulosis of the sigmoid colon and a cholelithiasis, which seemed to have caused a chronic inflammation judging by the most likely inflammatory adhesion of the greater omentum onto the gallbladder (Fig. 1).

**Fig. 2:**
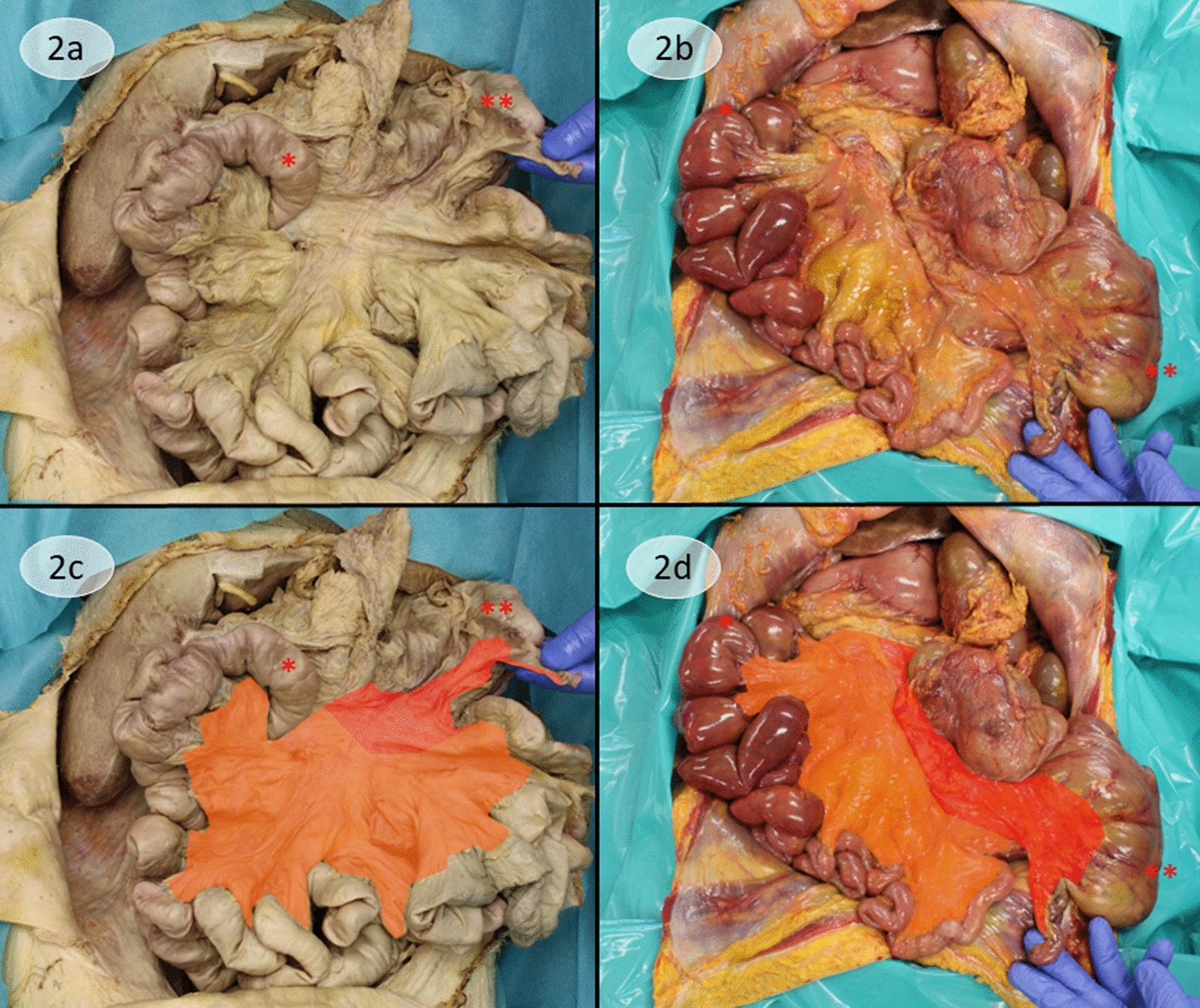
View of the malrotated loops with full display of the mesenteric root and comparison with the Cattell-Braasch maneuver. **a** Display of the unwound bowel without the typical frame of the colon around the small intestine due to the malrotation. The beginning of the jejunal loop (*) is in the upper right quadrant after the crossing of the duodenojejunal loop behind the mesenteric root to the right hemiabdomen (refer to Fig. 1b). The cecum (**) marking the beginning of the large intestine is in the upper-left quadrant; the attached appendix is manually displayed. **b** Display of the unwound bowel after Cattell-Braasch maneuver, showing a similar anatomy as in **a**. Here, the Treitz ligament was released, which allows a full derotation and transposition of the duodenum to the right hemiabdomen, therefore the beginning of the jejunal loop (*) is also in the upper right quadrant. Cecum (**) is visible in the left hemiabdomen. **c**, **d** The mesenteric root, being the remnant of the dorsal mesogastrium, is on full display, and the colors mark which part of the bowel the mesogastrium belongs to. Orange: dorsal mesogastrium of the small intestine; red: dorsal mesogastrium of the large intestine

The small intestine was located in the right hemiabdomen. The duodenum crossed the midline to the left and was fixated by Treitz ligaments; the duodenojejunal loop however immediately crossed back to the right hemiabdomen behind the mesenteric root. The large intestine was located in the left hemiabdomen with the cecum in the upper-left quadrant, clearly identified by the attached appendix. The parts of the large intestine up to the left colic flexure were mobile and attached to the back wall of the abdomen via the mesenteric root. The anatomy of the descending colon is consistent with normal conditions with a regular fusion with the abdominal wall. This state prompted the diagnosis of an atypical malrotation of the gut with partial rotation of the duodenum [8, 9]. A full view on the malrotated loops and the mesenteric root is given in Fig. 2.

**Fig. 3:**
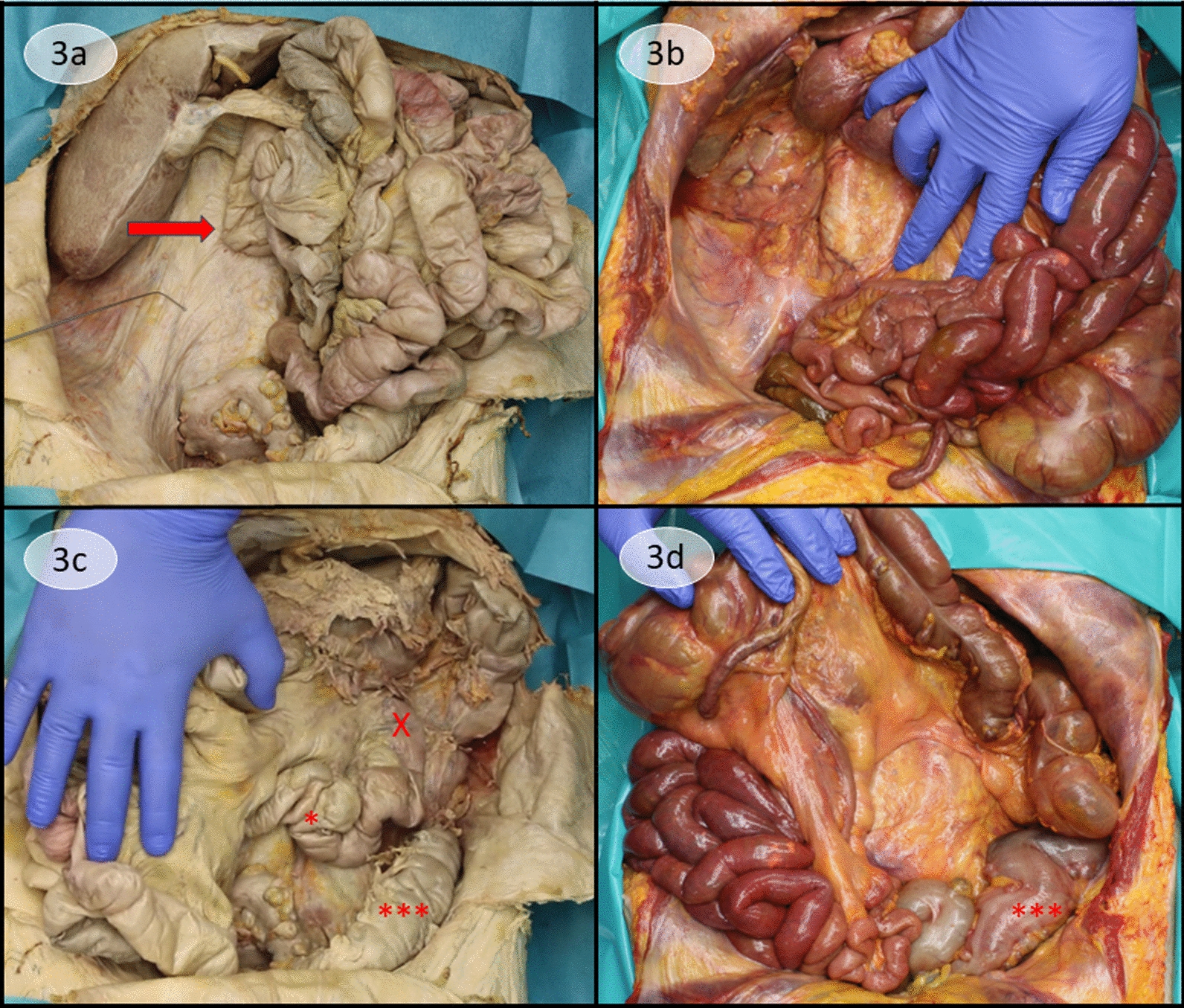
View on the back wall of the abdomen. **a** Display of the right fascia of Gerota, confining the retroperitoneum after dislodging the small intestine to the left. Instrument points at the smooth fascia of Gerota. Partially rotated duodenum (arrow) crosses the midline to the left. **c** After crossing the midline, the duodenojejunal flexure (X) makes another turn and the duodenojejunal loop (*) crosses the midline behind the mesenteric root to the right hemiabdomen (refer to Fig. 1b). The descending colon (***) shows no divergence from the usual topography. **b** Display of the right fascia of Gerota after Cattell-Braasch maneuver and thereby mobilization of the ascending colon. Duodenum is already mobilized by Kocherization and release of the ligament of Treitz, thereby allowing a full derotation. Therefore, the duodenal loop is not attached to the back wall anymore and lifted off the fascia of Gerota in this image. **d** Similarly, since the duodenum, as well as the duodenojejunal flexure, was mobilized and derotated, demonstration of the left hemiabdomen after pushing the bowel to the right shows no duodenum or duodenojejunal loop. Descending colon (***) shows no divergence from the usual topography

As presented in Fig. 3, shifting the bowel to the left side allowed free view onto the right fascia of Gerota, which sealed off the retroperitoneum. The surface was completely smooth and unscarred, the only adhesions being the usual duodenal fusion with the abdominal wall and fascia of Gerota. Shifting the small intestine to the right allowed a view onto the described duodenojejunal loop and descending colon.

## Discussion and conclusions

In this case report, we present an “atypical malrotation” of the gut with a partially rotated duodenum, thereby following the classification of Mehall et al. and the nomenclature used by Xiong et al. [8, 9]. Our case would stem from a lack of rotation beyond the very first stage, in which the primitive gut rotates 90°, which places the small intestine in the right hemiabdomen and the large intestine in the left hemiabdomen. The partial “rotation” of the duodenum is caused by duodenal proliferation, which pushes it behind the mesenteric root and over the midline, before the duodenojejunal loop returns to the right hemiabdomen instead of the usual position in the left hemiabdomen within the frame of the colon [4, 6, 13] (Fig. 1).

This anomaly is an unusual and also invaluable finding for manifold reasons. Firstly, the lack of rotation in itself—especially to the presented extent—is a rare occurrence. Secondly, the malrotation had no surgical implication during the lifetime of the body donor despite the old age, providing us with an unmarred abdominal cavity to study. Thirdly, this anatomy validates the surgical Cattell-Braasch maneuver, which is used to derotate the bowel in the adult for the purpose of a better overview and mobilization of the bowel for various surgical procedures [10]. This essentially reverts the embryologic development.

We however use the Cattell-Braasch maneuver in our cadaver studies to simplify the topography and enable us to study the remnants of the dorsal mesogastrium of the adult intestinal tube and fascial borders [11]. We focus specifically on those borders in order to transfer our insights to surgical oncology. But, how does a faultily rotated bowel help us understand fascial borders? And how do those fascial borders facilitate surgical tumor resection?

As we previously described [11], our group strongly favors the notion that thorough tumor resection is only possible if the tumor is removed along with the compartment in which it originates. This compartment is defined by a common embryologic origin with an enveloping layer of connective tissue. These layers enable the surgeon to preserve neighboring tissue that does not belong to the compartment that has to be eliminated, even if this tissue is in immediate vicinity to the region at risk.

This approach led to the creation of revolutionary methods such as the “total mesorectal resection” (TME) by Heald and the “total mesometrial resection” (TMMR) by Höckel [14, 15].

In order to separate the compartments, we previously revisited the Cattell-Braasch maneuver, since it enables the reconstitution of the much simpler embryologic anatomy [10, 11]. By doing this and reverting adhesions, we can separate layers that are merely artificially connected, but not due to their mutual ontogenetic origin. These layers can often be separated even by blunt dissection. We thereby unmask fascial borders that can lead the surgeon. However, our methodology carries the risk of preparational bias, which in turn provokes a comprehensible skepticism toward the results of such study.

Therefore, we were enthusiastic to see a situs in which we can trace how the bowel would be situated prior to a proper rotation and masking of fascial borders, which is exactly the state we manually restore via the Cattell-Braasch maneuver [11], except for the still fixed Treitz ligament in the malrotated specimen. In Figs. 2 and 3, we present an example of our Cattell-Braasch maneuver in a cadaver and compare it directly with the anatomy that we encounter in the malrotated specimen. Major analogies are the facilitation of the presentation of the remnants of the dorsal mesogastrium (Fig. 2) and the preservation of the fascia of Gerota as a barrier toward the retroperitoneum (Fig. 3). The confining fascia appears entirely immaculate when there is no fusion with the large intestine. At first, the distinction between the compartment of the intestine and the retroperitoneum might appear obvious. But the demonstration of the intact surface of the fascia of Gerota as in our malrotated specimens puts emphasis on the barrier that the fascia can pose. This requires an approach that respects borders and facilitates embryologic knowledge to find the proper layer to operate in.

## Conclusion

In this case report, we highlight an “atypical malrotation” from two angles.

From a practical clinical standpoint, the presented anatomy can remind the clinician of pitfalls while interpreting symptoms of a patient. We want to give clinicians an insight into the untouched anatomy of a malrotated bowel. We show that knowledge in embryologic development can still be relevant when encountering this incredibly rare anomaly even in older patients.

Additionally, we want to use this anomaly to validate a paradigm shift that puts fascial borders into the spotlight of surgery. We consider specimens that lack developmental steps to be a chance to explore borders and surfaces that very often vanish owing to fusion and adhesion. However, these are the borders that we deem essential for successful surgery. We can thereby prove that the anatomical conditions created in our previous studies with the Cattell-Braasch maneuver are not caused by bias in our dissection but that the presented borders and layers exist in a living person and are in plain sight if not masked by the normal developmental steps.

## Data Availability

All uncropped images are available from the corresponding author on reasonable request.
